# AAV-KLF7 Promotes Descending Propriospinal Neuron Axonal Plasticity after Spinal Cord Injury

**DOI:** 10.1155/2017/1621629

**Published:** 2017-08-13

**Authors:** Wen-Yuan Li, Ying Wang, Feng-Guo Zhai, Ping Sun, Yong-Xia Cheng, Ling-Xiao Deng, Zhen-Yu Wang

**Affiliations:** ^1^Department of Anatomy, Basic Medical College, China Medical University, Shenyang 110122, China; ^2^Department of Anatomy, Mudanjiang College of Medicine, Mudanjiang 157011, China; ^3^Department of Pharmacy, Mudanjiang College of Medicine, Mudanjiang 157011, China; ^4^Department of Pathology, Mudanjiang College of Medicine, Mudanjiang 157011, China; ^5^Spinal Cord and Brain Injury Research Group, Stark Neurosciences Research Institute, Indiana University School of Medicine, Indianapolis, IN 46202, USA

## Abstract

DPSN axons mediate and maintain a variety of normal spinal functions. Unsurprisingly, DPSN tracts have been shown to mediate functional recovery following SCI. KLF7 could contribute to CST axon plasticity after spinal cord injury. In the present study, we assessed whether KLF7 could effectively promote DPSN axon regeneration and synapse formation following SCI. An AAV-KLF7 construct was used to overexpress KLF7. *In vitro*, KLF7 and target proteins were successfully elevated and axonal outgrowth was enhanced. *In vivo*, young adult C57BL/6 mice received a T10 contusion followed by an AAV-KLF7 injection at the T7–9 levels above the lesion. Five weeks later, overexpression of KLF7 was expressed in DPSN. KLF7 and KLF7 target genes (NGF, TrkA, GAP43, and P0) were detectably increased in the injured spinal cord. Myelin sparring at the lesion site, DPSN axonal regeneration and synapse formation, muscle weight, motor endplate morphology, and functional parameters were all additionally improved by KLF7 treatment. Our findings suggest that KLF7 promotes DPSN axonal plasticity and the formation of synapses with motor neurons at the caudal spinal cord, leading to improved functional recovery and further supporting the potential of AAV-KLF7 as a therapeutic agent for spinal cord injury.

## 1. Introduction

The compromised ability of central nervous system neurons to regenerate their axons after injury is a well-documented deficit and a significant hindrance to post-SCI therapeutic strategies. Due to a variety of microenvironmental and endogenous molecular cues, axonal plasticity and synaptic recovery after SCI remain suboptimal, even after endogenous injury-repair mechanisms are considered. The sheer number of genes that must be up- or downregulated to reinitiate axon extension after injury presents a major challenge toward targeting the intrinsic neuronal growth state. One possible solution is the manipulation of underlying transcription factors (TFs) in injured neurons to drive the expression of downstream regeneration-associated genes (RAGs) [4]. A growing number of TFs have been functionally linked to axon growth in a variety of cell types. For example, neuroregulin 1 has been recently demonstrated as a crucial regulator of Schwann cell differentiation and remyelination in the injured spinal cord and thus as an attractive candidate for enhanced endogenous repair strategies [5]. Another study in a model of incomplete SCI reported that neurotrophic upregulation demonstrated an injury-specific BDNF response including regenerative sprouting and increased connectivity of injured neurons with propriospinal interneurons [6]. Other promising targets include the PRG3 gene, which induces filopodia formation and axonal growth and has also been shown to overcome a range of neurite growth inhibitors, though not yet evaluated in SCI specifically [7].

KLF7 is a transcription factor that has been found to play a role in cell fate specification of neuroectodermal and mesodermal cell lineages, regulating neuron markers and nerve growth factors such as NGF and TrkA [8]. Studies from optic nerve injury in zebrafish have also described KLF7 as a regulator of axon outgrowth [9]. Moreover, overexpression of KLF7 has demonstrated the ability to promote sprouting and axon regeneration in the adult corticospinal tract (CST) [10]. In peripheral nerve injury, KLF7 upregulation strategies have supported that KLF7 promotes axonal regeneration in injured nerves and that it acts as a growth-promoting transcription factor in injured neurons [11]. Similarly, when combined with chondroitinase viral expression, KLF7 promoted the regenerative growth of CST axons to the injury site in a model of spinal injury [12], though singularly, KLF7 upregulation was not apparently sufficient to induce axonal regeneration.

Descending propriospinal neurons (DPSNs) are particularly important in locomotion. As such, in SCI, restoring or maintaining the functional integrity of DSPNs and their axons may be a crucial component of motor functional recovery. T9 contusion models have reported up to a 23% loss of long descending propriospinal tract neurons as detected by retrograde labeling [13]. They are attractive candidates for SCI due to their demonstrated ability to form new intraspinal circuits [14] and act as relays between cortical and spinal targets after injury (Bareyre et al. 2004). For example, in rat spinal cord hemisections, neurochemical stimulation of propriospinal neurons enhanced locomotor-like activity when coupled with brainstem activation [15], suggesting that it plays a critical role in transmission of locomotor command signals. Further, in a lamprey injury model, DPSNs have even been suggested as indirect activators of spinal locomotor networks in the absence of axonal regeneration of injured neurons [16]. This cell population becomes even more relevant in light of the observation that propriospinal axonal regeneration is more easily achieved by therapeutic methods than supraspinal regeneration [17]. Moreover, DPSNs appear to be more reactive toward regenerative treatment [18, 19].

All things considered, the effectiveness of KLF7 upregulation strategies after SCI has not yet been evaluated in the critical DPSNs. Thus, to assess whether KLF7 could effectively promote DPSN axon plasticity and synapse formation, we modeled SCI using T10 contusion in young adult mice and examined KLF7 and KLF7 gene targets following the delivery of AAV-KLF7 to the T7–9 levels above the injury site. DPSN morphological and electrophysiological traits were evaluated along with functional outcomes. We report that AAV-KLF7 overexpression upregulated KLF7 target genes NGF and TrkA and enhanced neurite outgrowth in both spinal cord neurons and DRG neurons *in vitro*. An AAV-KLF7 injection into the T7–9 levels above the lesion was also found to improve the electrophysiological and morphological characteristics of DPSNs at the caudal spinal cord, consequently improving functional parameters. These findings confirm the therapeutic effectiveness of KLF7 overexpression in axonal plasticity and synaptic plasticity in a motor-relevant neuronal population, furthering the case for AAV-KLF7 as a therapeutic agent in spinal cord injury.

## 2. Materials and Methods

### 2.1. Viral Vector Preparation

The AAV-GFP virus contained both AAV serotype 2 capsid and expressed GFP. GFP expression which was expressed under the control of the cytomegalovirus (CMV) immediate-early promoter (AAV2-GFP, 1.0 × 10^13^ viral particles/ml; Vector BioLabs, Philadelphia, USA) was used as a control. Mouse KLF7 was subcloned into an AAV2 vector cassette under the control of the CMV (AAV-m-KLF7; Vector BioLabs, Philadelphia, USA).

### 2.2. Rat E15 Primary Spinal Cord Neuron Culture

Sprague-Dawley rat embryos (E15) were used for isolation of spinal cord neurons according to a previously established protocol [20]. Briefly, rat E15 spinal cords were harvested and placed in L15 medium. The dissociated cells were washed with and triturated in 10% heat-inactivated fetal bovine serum (FBS), 5% heat-inactivated horse serum (HS), and 2 mM glutamine DMEM (all culture reagents were obtained from Gibco, USA) and cultured in a 10 cm plate for 30 min at 37°C to eliminate glial cells and fibroblasts. Neurons were next plated onto poly-L-lysine-coated 48-well plates and incubated in a humidified atmosphere containing 5% CO_2_ at 37°C with 10% FBS + 5% HS + 2 mM glutamine DMEM for 16 hours. Medium was then replaced with serum-free neurobasal medium supplemented with 2% B27 (Gibco), 1% N2 (Gibco), and 2 mM glutamine. All experiments were performed between 7 and 10 days of plating.

### 2.3. AAV-KLF7 Transduction in Spinal Cord Neurons *In Vitro*

Spinal cord neurons were seeded on 6-well plates (5 × 10^5^ cells/well) and grown to over 90% confluence. After confluence was reached, cells were pretreated with 4–6 *μ*g/ml polybrene (Sigma, USA) for 30–60 min followed by infection with AAV2-GFP (final concentration 6.5 × 10^9^ viral particles/ml) and AAV2-KLF7 (6.5 × 10^9^ viral particles/ml) at a multiplicity of infection (13,000 moi). Four days after virus introduction, infection media were replaced with fresh media and cells were collected for immunostaining, Western blot, and transplantation experiments. For cells undergoing immunostaining procedures, cultures were fixed and examined using a mouse monoclonal anti-*β*-III-tubulin antibody (1 : 200; Sigma, St. Louis, USA), KLF7 (1 : 200; Novus Biologicals, Colorado, USA), and DAPI (1 : 200; Sigma, St. Louis, USA) to identify neurite outgrowth and confirm KLF7 expression.

### 2.4. Dynamic Neurite Outgrowth *In Vitro*

Spinal cord neurites were seeded at 2 × 10^5^ per well in 48-well plates four days after viral infection. The dynamic outgrowth of neurites was recorded using the IncuCyte ZOOM Kinetic Imaging System (Essen BioScience, USA) every 4 hours for 4 days after viral treatment. Three views from each well were recorded and data were analyzed by IncuCyte ZOOM software according to a previous methodology [21] (*n* = 50 neurons/group).

### 2.5. DRG Explant Cultures

Lumbar dorsal root ganglia (DRG) were extracted from Sprague-Dawley rat embryos at E15 and placed in 12-well dishes. 600 *μ*l of conditioned medium collected from confluent AAV-GFP or AAV-KLF7 cultures (collected at various 24 h intervals) or medium supplemented with BDNF (730 ng/ml) and NT-3 (730 ng/ml) was added to the wells for 3 d at 37°C in 5% CO_2_. DRG explant cultures were subsequently fixed and immunostained using mouse monoclonal anti-*β*-III-tubulin antibody (1 : 200; Sigma, St. Louis, USA). Neurite outgrowth was evaluated by Sholl analysis according to a previous study [22]. Briefly, we used grids of concentric circles 10 mm apart and counted the average neurite length and neurite length in angle bins.

### 2.6. Animals

Thirty-six C57BL/6 mice (male = 18, female = 18, eight weeks old, weight range 18–22 g) were obtained from the Experimental Animal Center of China Medical University (Certification number SCXK Liao 2003-0009). Animals were housed with three or four mice per cage and received standard food and water ad libitum. All experimental protocols used in this study were reviewed and approved by the Animal Care and Use Committee of China Medical University.

### 2.7. *In Vivo* Experimental Groups and Surgical Procedures

**Figure 1:**
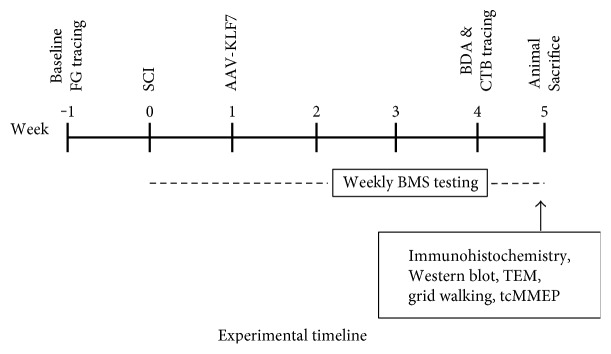
Experimental timeline. SCI: spinal cord injury; AAV: adeno-associated virus; CTB: cholera toxin B; FG: Fluoro-Gold; tcMMEP: transcranial magnetic motor-evoked potentials; BDA: biotinylated dextran amine; BMS: Basso Mouse Scale; TEM: transmission electron microscope.

Mice were randomly divided into three groups—group I: sham group (*n* = 12); group II: SCI + AAV-GFP group, contusive SCI and injection with AAV2-GFP control vector (*n* = 12); and group III: SCI + AAV-KLF7 group, contusive SCI and injection with AAV2-KLF7 (*n* = 12) (Figure 1).

Mice were anesthetized with a xylazine (10 mg/kg) and ketamine (100 mg/kg) cocktail delivered by intraperitoneal injection. A contusive SCI was performed after mice were fully anesthetized at the T10 level using the Louisville Injury System Apparatus (LISA) [23]. Viral injections occurred 1 week after SCI; mice in the SCI + AAV-GFP group and SCI + AAV-KLF7 group received a 0.5 mm displacement contusion at a velocity of 1.0 m/s while sham animals received laminectomy only. Microinjections of KLF7 were then delivered into the T8-9 level of the mouse spinal cord according to a previously described method [24]. Briefly, a beveled glass micropipette (external diameter, 10–20 *μ*m) was loaded with 2 *μ*l of AAV-KLF7 (6.5 × 10^9^ viral particles/ml) or AAV-GFP (6.5 × 10^9^ viral particles/ml) and bilaterally injected into the spinal cord (2 *μ*l/side) at 0.6 mm lateral to the midline and 1.5 mm ventral to the dorsal surface of the cord for 10 min. The overlying musculature was closed using sutures, and the skin was closed using wound clips after which animals were treated with Marcaine at the incision site.

Mice were placed on a heating blanket and maintained at 37°C until completely recovered from anesthesia. All mice were housed and fed routinely and monitored closely for changes in their general conditions and locomotor capacity.

### 2.8. Histological Assessments

Five weeks after contusion injury, animals were perfused and the cord segments were dissected, embedded, and sectioned in 20 *μ*m increments according to our previously established protocol [24]. A set of serial cross sections of the spinal cord was stained for myelin using Luxol fast blue and was counterstained with cresyl violet-eosin. Lesion areas were outlined and quantified using an Olympus BX60 microscope equipped with a Neurolucida system (MicroBrightField, Colchester, VT). Areas of demyelination were quantified using ImageJ (NIH imaging software, Bethesda, USA).

### 2.9. Immunohistochemistry

Before sectioning, the cords were segmented using the dorsal roots as landmarks as follows: serial, 25 *μ*m thick sections were cut from the rostral spinal cord (T7–9 segment), epicenter (the injured T10 segment), and caudal spinal cord (L2–L5 lumbar segment) using a cryostat. The images of every 5th section were quantified.

Sections were carefully mounted onto Superfrost Plus slides (Menzel-Glaser, Braunschweig, Germany) and processed for immunohistochemical detection. Tissues were stained with antibodies against KLF7 (1 : 200; Novus Biologicals, Colorado, USA), peripheral myelin or P0 (1 : 200; Sigma-Aldrich, Missouri, USA), GFAP to identify astrocytes (1 : 200; Sigma-Aldrich), BDA to identify astrocytes (1 : 200; Sigma-Aldrich), SYP to identify presynaptic components (1 : 500; Sigma-Aldrich), CC1 to identify oligodendrocytes (1 : 200; Sigma-Aldrich), and NG2 to identify oligodendrocyte precursors (1 : 200; Sigma-Aldrich). The immunoreactivity signals were visualized by goat anti-rabbit IgG (FITC), anti-mouse IgG (TRITC), and anti-donkey IgG (CY5) (1 : 200; Jackson ImmunoResearch Laboratories, Pennsylvania, USA), and images were obtained with an Olympus BX-60 epifluorescent microscope and Neurolucida software (MicroBrightField). The cross-sectional area was measured using an Olympus BX60 microscope at a 20x view field (27,000 *μ*m^2^), and pixel intensity was measured on images with a uniform exposure setting and analyzed using ImageJ (NIH, Bethesda, MD). Five sections were analyzed per sample. No image modification was performed prior to measurements. Multipanel figures were assembled in Adobe Photoshop CS6 software (Adobe Systems, San Jose, CA).

All immunohistochemistry image quantifications were performed blind. The numbers of retrogradely labeled neurons were counted from every eighth section in each spinal block through the microscope eyepiece by an experimenter blinded to the experimental condition. Only neurons with a nucleus and proximal dendritic processes that were visible upon focusing through the thickness of the section were included in the cell counts. Cell counts are reported from each spinal block as group means. To analyze propriospinal axon sprouting and synapse formation, contacts onto motor neurons throughout the lumbar level of the sectioned spinal cord were used for quantification. Propriospinal axon sprouting was in some cases defined as projections or branches of axons, cholera toxin B-labeled motoneurons, or SYP-labeled synapses in the ventral gray of the L2–L5 spinal cord using triple immunohistochemical staining. Axonal sprouting was quantified by counting the pixel intensity of BDA-positive axonal sprouts surrounding CTB-positive motoneurons in the ventral gray (VIII–IX) using ImageJ [25]. To analyze the synapse contacts onto motor neurons, cholera toxin B-labeled motoneurons were automatically selected and a constant threshold was used to segment and obtain an estimated average density for each label using ImageJ software [26]. Detection of the immunoreactivity was performed using a fixed threshold for every staining (the motoneurons and dendrites were labeled with CTB and synapses were labeled with SYP). Immunoreactivity was evaluated in a circular perimeter of 5 *μ*m width surrounding each motoneuron. This 5 *μ*m width perimeter covered the synaptic area surrounding motoneurons, limiting overlap with the synapses of neighboring motoneurons.

### 2.10. Electron Microscopy

Spinal cord tissue from the ventral white matter was obtained from a normal uninjured spinal cord (sham group) as well as from the lesion around the epicenter in SCI + AAV-GFP and SCI + AAV-KLF7 groups; these segments were fixed overnight in the solution containing 2% glutaraldehyde and 5% sucrose in 0.1 M sodium cacodylate buffer (pH 7.4). The following day, 1 hr incubation was performed in 1% osmium tetroxide in the same buffer. The tissue was then embedded in Spurr's epoxy resin and cured at 70°C. Ultrathin sections (70–90 nm) were collected on copper mesh grids (600 bars per inch) and subsequently counterstained with 4% uranyl acetate in 50% ethanol and Reynolds' lead citrate. Finally, sections were examined using a Philips CM10 electron microscope. For each mouse, micrographs representing different grid squares of a single section were randomly selected for quantitative analysis. The total number of axons was obtained with Stereo Investigator software (MicroBrightField). Myelinated axons were identified by their characteristic myelin sheaths relative to the diameter of the axons. Unmyelinated axons were identified by the absence of the characteristic myelin sheaths. Myelin ratio (MR) was used to detect whether AAV-KLF7 could promote axonal myelination around the lesion site following SCI as previously described [27].

We measured the axonal diameter (d) as the shortest distance across the center of axons, avoiding the myelin sheath thickness. The axonal diameter plus the total myelin sheath thickness on both sides was defined as fiber diameter (D). The myelin ratio (MR) was calculated using the D/d ratio. For each mouse, micrographs representing different grid squares of a single section were randomly selected for quantitative analysis. More than 50 randomly chosen fibers per animal were analyzed, using the MIAS image analysis system by two blinded researchers.

### 2.11. Anterograde and Retrograde Tracing

On day 28 after spinal cord injury, after animals were anesthetized, bilateral and stereotaxic injections of biotinylated dextran amine (BDA; 10%, 1 *μ*l/site; Molecular Probes) were made into the intermediate gray matter of the T7-T8 cord at distances of 3–6 mm rostral to the contusion (for BDA, 1 injection/site/mm) as described in a previously published work (*n* = 8/each group) [18].

BDA staining was subsequently performed. Specifically, slides were dried at 38°C for 1 h and washed twice for 10 min in 50 mM Tris-buffered saline (TBS, pH 7.4), followed by two 45 min washes with TBS containing 0.5% Triton X-100. Slides were then incubated overnight with an ExtrAvidin®-FITC buffered aqueous solution or ExtrAvidin-TRITC buffered aqueous solution (1 : 200; Sigma, St. Louis, USA) according to the manufacturer's instructions. The reaction was monitored closely and stopped by extensive washing in water.

For the retrograde tracing, both sides of animal sciatic nerves were exposed and 1-2 *μ*l of 2% solution of Alexa Fluor-labeled (594) cholera toxin B (Invitrogen, USA) was injected using a Hamilton syringe with a 30-gauge needle. CTB retrograde labeling targeted the lumbar motor neurons (*n* = 8/each group).

### 2.12. FG Retrograde Labeling of DPSNs

Before one-week surgery, for Fluoro-Gold (FG) retrograde labeling, 2% FG (Sigma39,286, Sigma-Aldrich, St. Louis, MO) was bilaterally injected into the intermediate gray matter (laminae V–VII) of the L2 spinal cord (0.75 *μ*l/side) using a stereotaxic apparatus [18]. Analysis of the DPSN was restricted to the T6–T10 spinal cord segments, with axonal projections to the L2 spinal cord segment. Additionally, only neurons whose somata were located in the intermediate gray matter (Rexed lamina VII) were included for analysis.

### 2.13. Western Blotting and PCR

Spinal cord neuron cell samples were homogenized and processed for Western blot analysis 4 d after viral transfection with evaluated AAV-KLF7 that significantly increased the expression of KLF7 and target proteins *in vitro* (6 wells/each group). To evaluate changes in KLF7 expression at the thoracic level *in vivo*, samples collected at different time points (1 day and 1, 2, 3, and 4 weeks after SCI) were homogenized for total protein extraction (*n* = 3). To evaluate the expression of KLF7 target proteins at the thoracic level, the spinal cord was harvested five weeks after SCI (*n* = 6). The spinal cord was dissected and washed in saline. After the dura mater was carefully removed, the tissue was snap-frozen on dry ice. Next, protein isolation was performed and protein samples (20 *μ*g) were electrophoresed on SDS-polyacrylamide gels and transferred to polyvinylidene difluoride membranes (Millipore, Bedford, MA). The blots were incubated with primary antibodies against KLF7 (1 : 500; Novus Biologicals, Colorado, USA), NGF (1 : 500; Sigma, USA), TrkA (1 : 1000; Sigma, USA), GAP43 (1 : 1000; Sigma-Aldrich, Missouri, USA), and P0 (1 : 1000; Sigma, USA) overnight followed by incubation with an HRP-conjugated secondary antibody for 1 h at room temperature (1 : 5000). Blots were visualized using the enhanced chemiluminescence (ECL) plus the detection system (GE Healthcare, Little Chalfont, UK); the exact exposure time was determined as 1 minute and used uniformly across all proteins. Experiments were repeated three times under the same condition; for determination of individual band differences, the intensity of each band was calculated from ImageQuant and the ratio of the target protein to the housekeeping protein GAPDH was generated. Data were expressed as mean ± standard deviation (SD) of the mean. Error bar is SD (standard deviation). Using one-way ANOVA with Tukey's post hoc test to determine statistical significances, experiments were repeated three times under the same conditions for a representative average.

Using a similar dissection method, total RNA was extracted using the RNeasy Kit (Qiagen, California, USA) according to the manufacturer's instructions. KLF7, NGF, and TrkA cDNA was next amplified from 2.5 g total RNA using the SuperScript II First-Strand Synthesis RT-PCR kit (Life Technologies) and the following parameters: KLF7 reverse transcription at 42°C for 15 minutes using primers 5-TTTCCTGGCAGTCATCTGCAC-3 and 5-GGGTCTGTTTGTTTGTCAGTCTGTC-3, 219 bp; NGF primers 5-CATAGCGTAATGTCCATGTTGTTCT-3 and 5-CTTCTCATCTGTTGTCAACGC-3, 395 bp; and TrkA primers 5-ATGGACAACCCTTTCGAGTTCAAC-3 and 5-GACCCCAAAAGGTGTTTCGTCC-3, 412 bp. Finally, about 100 ng of purified genomic DNA was amplified for 35 to 50 cycles using the following parameters: 94°C for 2 min, 94°C for 1 min, 59°C for 1 min, and 72°C for 2 min, and genotyping was performed as previously described [28].

### 2.14. Muscle Weight and Motor Endplate Morphology

To assess potential atrophic changes in the target musculature after SCI, the right TA muscles were removed immediately after perfusion (5 weeks after injury) and weighed. Muscles were then postfixed overnight in the same fixative as was used for perfusion and then transferred to sucrose phosphate buffer (10% *w*/*v*, pH 7.4) solution for one week. Muscle tissues were then rinsed in distilled water, blocked into proximal and distal segments, and flash-frozen in 2-methylbutane. Muscle segments were then sectioned (45 *μ*m) longitudinally (for examination of the motor endplate area and density) on a cryostat. Muscle fibers were assessed after staining with Milligan's trichrome stain. Motor endplate size and density were assessed after staining for acetylcholinesterase using the Roots-Karnovsky method [29].

### 2.15. Measurements of Axonal Conduction

Nerve conduction was assessed using transcranial magnetic motor-evoked potentials (tcMMEP) as an *in vivo* electrophysiological measure of motor pathway function according to our previously outlined method [30]. The tcMMEP responses were elicited by activation of subcortical structures using an electromagnetic coil placed over the cranium. Action potentials descending in the ventral spinal cord and synapsing onto motoneuron pools were used to record output signals from both gastrocnemius muscles [31, 32].

### 2.16. Behavioral and Locomotor Assessments

The Basso Mouse Scale (BMS) locomotor test was performed 1, 2, 3, 4, and 5 weeks after AAV-KLF7 injections and contusive SCI by 2 independent observers lacking knowledge of the experimental groups according to a method published previously [33]. During evaluation, animals were allowed to walk freely on an open-field surface for 4 min sessions as observers scored the locomotor capacity of the animals. The BMS was observed by two observers who were blinded to the allocation of the animals. Videos were recorded for further analysis in case of divergence in the BMS scores assigned by the observers. If there was a divergence, the evaluators discussed the case until they reached a consensus.

Additionally, animals were placed to walk on a 1 m long horizontal runway of metal grid bars elevated 30 cm from the ground. A defined 10-bar sector was chosen for analysis. To prevent habituation to a fixed bar distance, the bars in this sector were irregularly placed (1–4 cm spacing) and were changed after every testing session. Analysis of grid walk included counting the number of errors in foot placing occurring during each bar sector trial. Each animal had to cross this sector three times. If the animal did not show hind limb weight support, it made two errors per bar, resulting in a total of 20 errors [34].

### 2.17. Statistical Analysis

The BMS performed a two-way repeated-measures ANOVA to compare the three groups. No normality tests were performed because the BMS is a nonlinear scale. Post hoc analysis at individual time point was performed with Tukey's test.

Statistical analysis was performed using SPSS software (version 13.0; SPSS, Illinois, USA). All values were expressed as the mean with the corresponding standard deviation. Sample sizes were determined using statistical software to calculate the minimum number of animals required for sufficient statistical power for each assay. All reported sample sizes are above the minimum calculated requirement.

The Kolmogorov-Smirnov test was used to distinguish between parametric or nonparametric data. All parametric values were analyzed using two-tailed Student's *t*-test or one-way ANOVA and Tukey's post hoc test. Analyses were carried out with GraphPad Prism 6.0 software. A *P* value of <0.05 was considered statistically significant.

## 3. Results

### 3.1. Efficacy of AAV2-KLF7 Gene Transfer to Spinal Cord Neurons *In Vitro*

**Figure 2:**
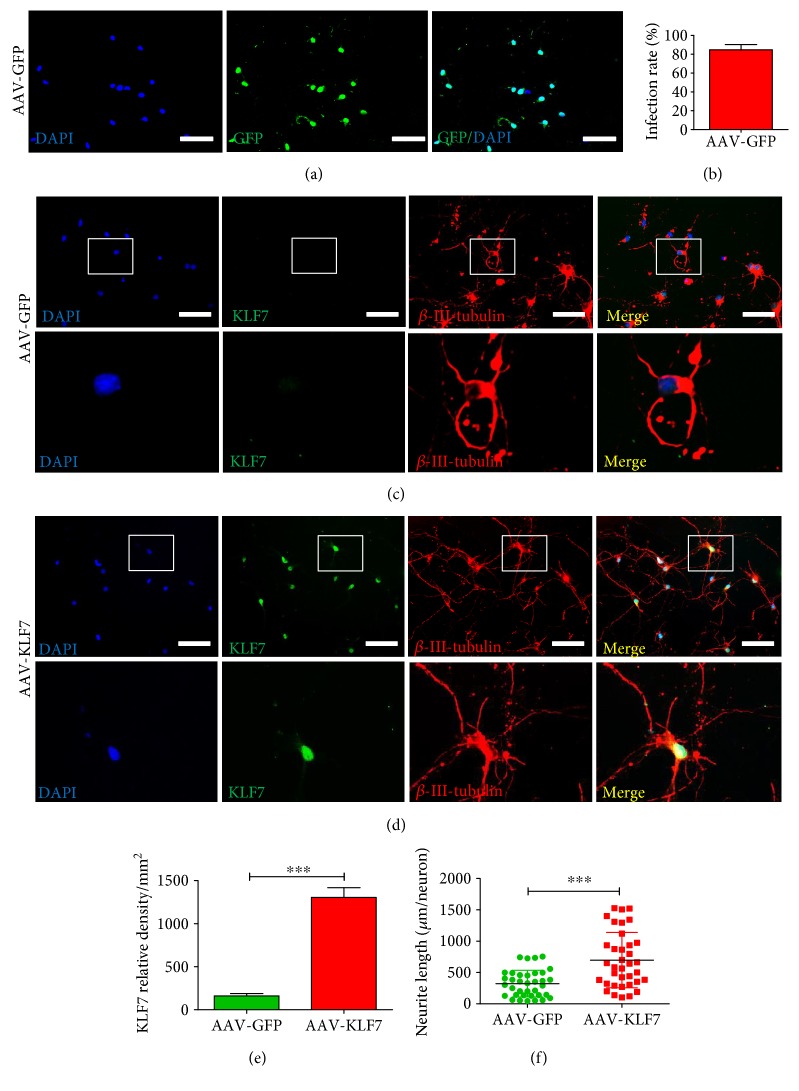
Efficacy of AAV2-KLF7 gene transfer to spinal cord neurons *in vitro*. (a) AAV-GFP-infected cultured spinal cord neurons. Scale bars: 50 *μ*m. (b) Quantitative analysis of AAV2-GFP infection rate. Representative immunohistochemical staining of KLF7 (green) and *β*-III-tubulin (labeling neurite and dendrite outgrowth, red) with nuclear staining (DAPI, blue) in cultured spinal cord neurons in the AAV-GFP (c) and AAV-KLF7 groups (d). Block shows the KLF7 colabeling of nuclei and neurite outgrowth. Quantitative analyses of the KLF7 relative density (e) and neurite length/neuron (f) in the two groups. *n* = 6 wells/group. Student's *t*-tests, ^∗∗∗^*P* < 0.001. Scale bars: 200 *μ*m.

To verify the successful transduction of KLF7 with the AAV2 vector, we first examined AAV2-GFP-infected spinal cord neurons *in vitro* (Figure 2(a)). The AAV2-GFP transfection of spinal cord neurons was successful, with a transfection efficiency of 84.70 ± 5.57% (Figure 2(b)). Next, the transfection efficiency of AAV2-KLF7 was similarly evaluated in spinal cord neurons using immunohistochemical staining of KLF7 (Figures 2(c) and 2(d)). We found that the expression of KLF7 was significantly increased following AAV2-KLF7 (1306 ± 275.1) transduction compared to that of the AAV-GFP (160.7 ± 68.73)-receiving group (*t*_10_ = 9.888, *P* < 0.001) (Figure 2(e)). Additionally, *β*-III-tubulin immunolabeling was used to evaluate whether induction of KLF7 promoted neurite outgrowth of spinal cord neurons. A significant increase was detected in neurite length average of the AAV-KLF7 group (697.6 ± 441.7 *μ*m/neuron) compared to AAV-GFP-transfected controls (322.8 ± 213.3 *μ*m/neuron) (*t*_70_ = 4.585, *P* < 0.001) (Figure 2(f)).

### 3.2. AAV-KLF7 Significantly Increased the Expression of KLF7, NGF, and TrkA in Spinal Cord Neurons *In Vitro*

**Figure 3:**
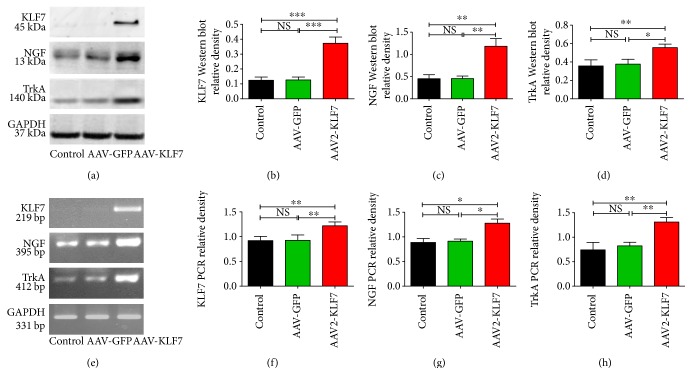
AAV-KLF7 significantly increased the expression of KLF7 and target proteins *in vitro*. (a) Representative Western blot analysis of KLF7, NGF, and TrkA expression in the cultured spinal cord neurons of the three groups is shown at 4 days *in vitro* (6 wells/each group). The graphs represent the relative density of KLF7 (b), NGF (c), and TrkA (d) protein. (e) Representative RT-PCR analysis of KLF7, NGF, and TrkA expression in the cultured spinal cord neurons of the three groups is shown at 4 days *in vitro* (*n* = 6). The graphs represent the relative density of KLF7 (f), NGF (g), and TrkA (h) mRNA. Error bars denote SD. ^∗^*P* < 0.05, ^∗∗^*P* < 0.01, and ^∗∗∗^*P* < 0.001. NS: not significant. One-way ANOVA, Tukey's post hoc test.

Four days after *in vitro* transfection with the AAV-KLF7, KLF7 protein (AAV2-KLF7, 0.372 ± 0.041; AAV-GFP, 0.126 ± 0.019; and control, 0.124 ± 0.021; *F*_2, 15_ = 70.63, *P* < 0.001) and mRNA (AAV2-KLF7, 1.218 ± 0.078; AAV-GFP, 0.925 ± 0.109; and control, 0.918 ± 0.086; *F*_2, 15_ = 13.68, *P* < 0.05) expression was found significantly increased compared to that of AAV-GFP controls (Figures 3(b) and 3(f)). Similarly, NGF protein (AAV2-KLF7, 1.181 ± 0.174; AAV-GFP, 0.454 ± 0.058; and control, 0.451 ± 0.089; *F*_2, 15_ = 37.91, *P* < 0.001) and mRNA (AAV2-KLF7, 1.278 ± 0.147; AAV-GFP, 0.912 ± 0.076; and control, 0.888 ± 0.136; *F*_2, 15_ = 9.293, *P* < 0.05) and TrkA protein (AAV2-KLF7, 0.556 ± 0.038; AAV-GFP, 0.377 ± 0.051; and control, 0.357 ± 0.065; *F*_2, 15_ = 12.79, *P* < 0.05) and mRNA (AAV2-KLF7, 1.310 ± 0.091; AAV-GFP, 0.823 ± 0.073; and control, 0.741 ± 0.151; *F*_2, 15_ = 23.20, *P* < 0.05) expression in the AAV-KLF7 group was considerably higher than that in control cells and AAV-GFP-infected cells (Figures 3(c), 3(d), 3(g), and 3(h)). However, no difference in protein or mRNA expression of KLF7, NGF, or TrkA was found between the control and AAV-GFP group. As NGF and TrkA are considered downstream targets of KLF7, these results suggested a successful and functional induction of KLF7 by AAV2 infection in spinal cord neurons *in vitro*. Due to group-specific increases in KLF7 and its targets, it is unlikely that increases can be attributed to intrinsic or compensatory phenomena.

### 3.3. KLF7 Overexpression Promotes Neurite Outgrowth of Spinal Cord Neurons and DRG Explants *In Vitro*

**Figure 4:**
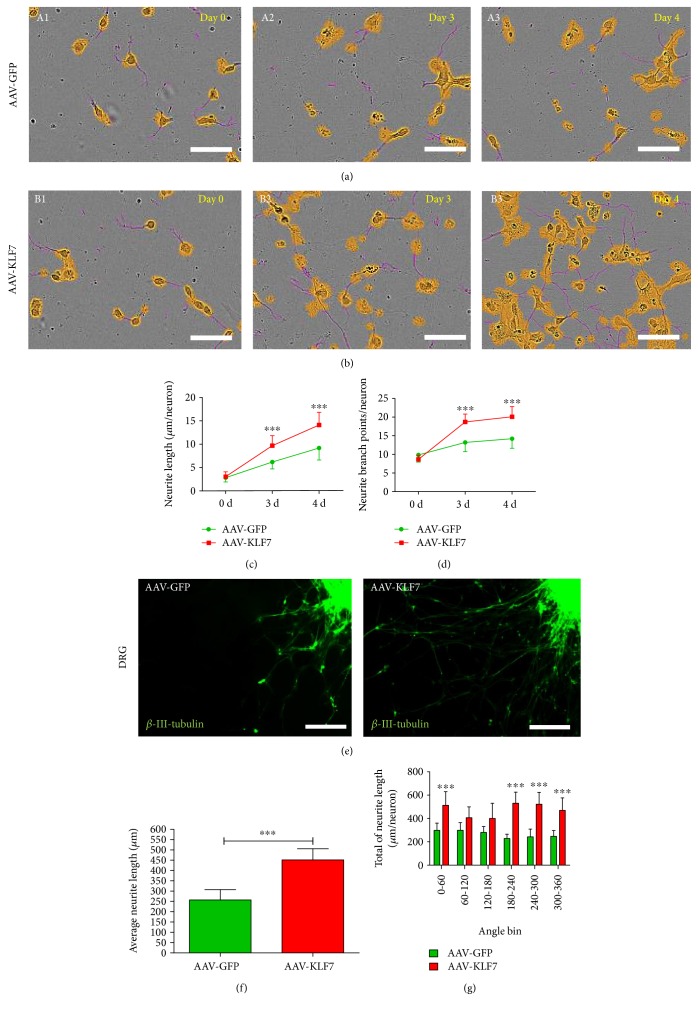
AAV-KLF7 promotes neurites outgrowth *in vitro*. Time-lapse imaging of spinal cord neuron axonal outgrowth in AAV-GFP (a) and AAV-KLF7 (b) groups on 0, 3, and 4 days (IncuCyte ZOOM Kinetic Imaging System) *in vitro*. Data represent neurite length/neuron (c) and neurite branch points/neuron (d) (*n* = 50 neurons/group). (e) Representative immunostaining of DRG explants in each group; neurite outgrowth was labeled with *β*-III-tubulin (green). A comparison of the average length (f) and total of neurite length per neuron within different angle bins (g) by Sholl analysis (*n* = 6/group). Error bars denote SD. ^∗∗∗^*P* < 0.001 versus the AAV-GFP control group. Student's *t*-tests. Scale bars: 50 *μ*m or 100 *μ*m.

To test the effect of AAV-KLF7 on neurite outgrowth of spinal cord neurons in real time, we used time-lapse imaging of spinal cord neurons using the IncuCyte ZOOM Kinetic Imaging System (Figures 4(a) and 4(b)). Results showed that compared to those of the AAV-GFP group, AAV-KLF7 neurons displayed dramatic increases in neurite length (3 d: AAV-GFP, 6.211 ± 1.490 *μ*m/neuron; AAV-KLF7, 9.723 ± 2.125 *μ*m/neuron; *t*_98_ = 4.236, *P* < 0.01; 4 d: AAV-GFP, 9.234 ± 2.575 *μ*m/neuron; AAV-KLF7, 14.162 ± 2.731 *μ*m/neuron; *t*_98_ = 2.993, *P* < 0.01 (Figure 4(c))) and neurite branching (3 d: AAV-GFP, 13.365 ± 2.490/neuron; AAV-KLF7, 18.736 ± 2.121/neuron; *t*_98_ = 7.934, *P* < 0.001; 4 d: AAV-GFP, 14.417 ± 2.570/neuron; AAV-KLF7, 20.110 ± 2.731/neuron; *t*_98_ = 12.58, *P* < 0.001 (Figure 4(d))) of spinal cord neurons 3-4 days after viral transfection.

Next, we evaluated the effect of KLF7 on neurite outgrowth of DRG explants using the same imaging techniques (Figure 4(e)). Sholl analysis showed that the average neurite length in the AAV-KLF7 group (451.70 ± 54.23 *μ*m) was significantly higher than the average neurite length in the AAV-GFP group (257.1 ± 49.95 *μ*m) (*t*_22_ = 7.921, *P* < 0.001) (Figure 4(f)). The AAV-KLF7 showed the greatest neurite length in 0–60° (512.8 ± 117.3 versus 297.8 ± 62.1 *μ*m, *P* < 0.05), 180–240° (530.1 ± 94.7 versus 228.5 ± 36.9 *μ*m, *P* < 0.05), 240–300° (522.3 ± 102.1 versus 242.3 ± 66.2 *μ*m, *P* < 0.05), and 300–360° (469.2 ± 106.6 versus 246.9 ± 49.7 *μ*m, *P* < 0.05) angle bins compared with the AAV-GFP (Figure 4(g)). Collectively, these results support that KLF7 overexpression plays an important role in promoting neurite outgrowth *in vitro*.

### 3.4. KLF7 Expression Is Dynamically Altered following SCI

**Figure 5:**
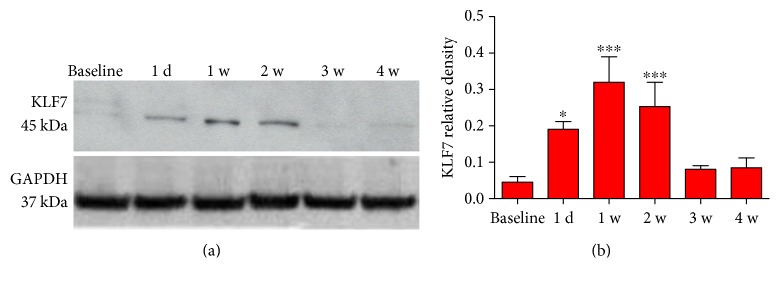
KLF7 expression is dynamically altered following SCI. (a) Western blot analysis revealed the endogenous change in KLF7 expression in the thoracic spinal cord injury tissue after SCI. (b) Graphs represent the KLF7 relative density of the bands normalized to GAPDH across different time points. *n* = 6, error bars denote SD. ^∗^*P* < 0.05, ^∗∗∗^*P* < 0.001 versus the baseline. One-way ANOVA, Tukey's post hoc test.

The expression changes in KLF7 following SCI were confirmed by Western blot protein analysis (Figure 5(a)). KLF7 protein levels were initially low in baseline, uninjured spinal cord tissue. The expression of KLF7 in the thoracic segments of the spinal cord was induced by 1 day post-SCI, peaking around 1-2 weeks postinjury and returning to baseline levels by 3-4 weeks (baseline: 0.015 ± 0.007; 1 d: 0.390 ± 0.021; 1 w: 0.420 ± 0.030; 2 w: 0.393 ± 0.015; 3 w: 0.126 ± 0.047; and 4 w: 0.021 ± 0.008; *F*_5, 24_ = 20.00, *P* < 0.001) (Figure 5(b)). The intrinsic injury response of the KLF7 protein supports a role for KLF7 in endogenous injury repair after SCI modeling *in vivo*.

### 3.5. AAV-KLF7 Significantly Increases the Expression of KLF7 and Its Downstream Targets and Mitigates Injury-Related Loss of the Myelination Protein following SCI

**Figure 6:**
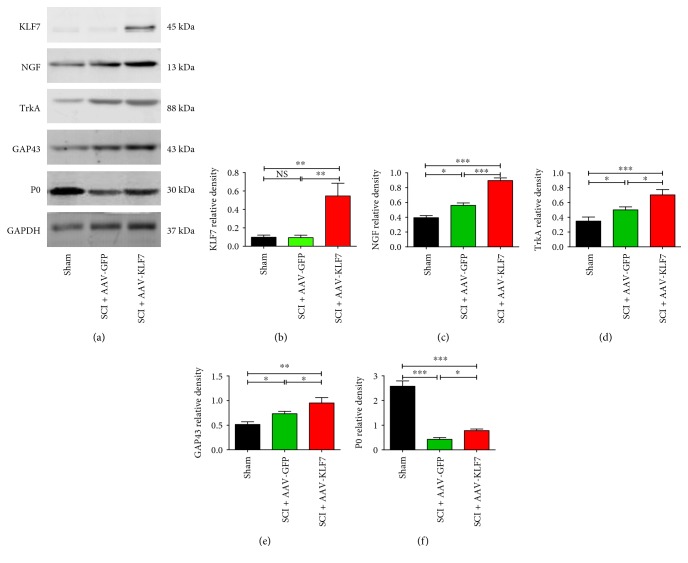
AAV-KLF7 significantly increased the expression of KLF7 and target proteins NGF, TrkA, GAP43, and P0 following SCI. (a) Representative Western blot analyses of KLF7, NGF, TrkA, GAP43, and P0 expression in thoracic spinal cord section tissues following SCI across three groups. The graphs for the analyses represent the relative density of KLF7 (b), NGF (c), TrkA (d), GAP43 (e), and P0 (f). *n* = 6 mice/group. Error bars denote SD. ^∗^*P* < 0.05, ^∗∗^*P* < 0.01, and ^∗∗∗^*P* < 0.001. NS: not significant. One-way ANOVA, Tukey's post hoc test.

To verify the successful delivery of AAV-KLF7 to the injured tissue, KLF7 expression was examined using Western blot protein analysis in thoracic segments of spinal cord tissues harvested 5 weeks postinjury (Figure 6(a)). In the sham (0.097 ± 0.023) and SCI + AAV-GFP groups (0.093 ± 0.026), only a small amount of KLF7 was detected. Meanwhile, animals receiving the AAV-KLF7 treatment (0.546 ± 0.138) demonstrated an increased amount of KLF7 protein expression comparatively (*F*_2, 15_ = 29.96, *P* < 0.001) (Figure 6(b)).

To test the effect of AAV-KLF7 on the expression of KLF7 target genes *in vivo*, we examined the expression of NGF and TrkA, the axon regeneration marker GAP43, and the myelinated axon marker P0 in thoracic spinal cord tissues (Figure 6(a)). The results demonstrated that injury upregulated the examined KLF7 protein targets and the axonal regeneration marker GAP43, after which AAV-KLF7-treated animals demonstrated higher expression in the thoracic segments of the injured spinal compared to AAV-GFP controls (Figure 6). Specifically, the NGF, TrkA, and GAP43 expression in the SCI + AAV-KLF7 group was higher than that in the SCI + AAV-GFP group (NGF: SCI + AAV-KLF7, 0.895 ± 0.064; SCI + AAV-GFP, 0.561 ± 0.055; and sham, 0.394 ± 0.047; *F*_2, 15_ = 61.25, *P* < 0.001; TrkA: SCI + AAV-KLF7, 0.703 ± 0.071; SCI + AAV-GFP, 0.500 ± 0.040; and sham, 0.348 ± 0.054; *F*_2, 15_ = 29.20, *P* < 0.001; and GAP43: SCI + AAV-KLF7, 0.950 ± 0.110; SCI + AAV-GFP, 0.732 ± 0.048; and sham, 0.513 ± 0.055; *F*_2, 15_ = 24.30, *P* < 0.01) (Figures 6(c), 6(d), and 6(e)). Additionally, the myelin maker P0 was drastically decreased after injury, likely due to demyelination and degeneration occurring after SCI. Treatment with AAV-KLF7, however, was able to significantly increase postinjury abundance of the P0 protein compared to AAV-GFP treatment (Figure 6(f); SCI + AAV-KLF7, 0.782 ± 0.063; SCI + AAV-GFP, 0.425 ± 0.077; and sham, 2.579 ± 0.216; *F*_2, 15_ = 280.2, *P* < 0.001). These results corroborate that the AAV-KFL7 upregulation technique was lasting and capable of impacting the downstream targets of the transcription factor including NGF-, TrkA-, and GAP43-mediated signaling cascades which have been shown to play roles in axon plasticity and remyelination. Additionally, treatment with KLF7 overexpression mitigated the injury-related decrease in the myelin protein P0.

### 3.6. AAV-KLF7 Treatment Improved Myelin Sparing following SCI

**Figure 7:**
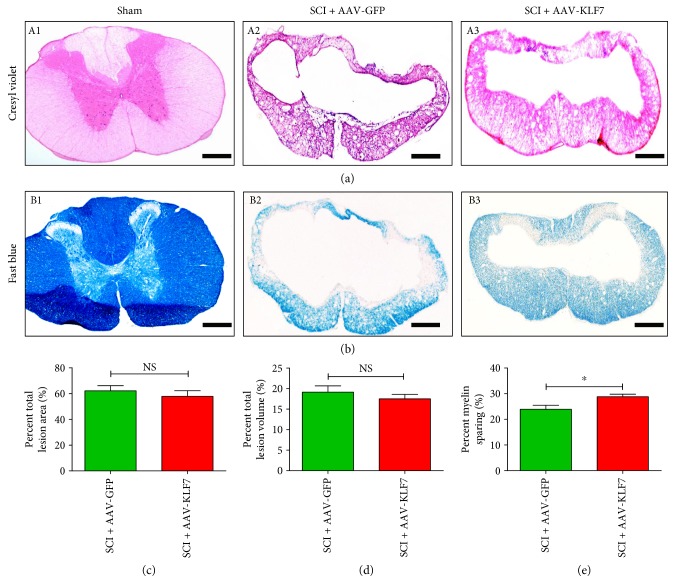
Histological comparison of AAV-KLF7- or AAV-GFP-treated groups five weeks after contusive SCI. Cresyl violet-eosin (a) and Luxol fast blue staining (b) in each group showed the level of SCI-induced tissue damage and loss of myelin. Quantitative analyses of the lesion area (c), lesion volume (d), and myelin sparing (e) (*n* = 6 mice/group). Error bars show SD. Student's *t*-tests, ^∗^*P* < 0.05. Scale bars: 300 *μ*m.

To further assess the role of AAV-KLF7 overexpression on myelination after injury and to examine the effects of the treatment on pathological features, the lesion area, lesion volume, and tissue sparing at the site of T10 contusion injury were evaluated by cresyl echt violet staining 5 weeks after contusive SCI. Results indicated that AAV-KLF7 treatment had no effect on the lesion area (SCI + AAV-KLF7, 57.78 ± 10.81%; SCI + AAV-GFP, 62.15 ± 9.68%; *t*_10_ = 0.737, *P* > 0.05) nor on the lesion volume (SCI + AAV-KLF7, 17.50 ± 2.44%; SCI + AAV-GFP, 19.14 ± 3.43%; *t*_10_ = 0.869, *P* > 0.05) (Figures 7(a), 7(c), and 7(d)) compared to the AAV-GFP treatment following SCI. However, Luxol fast blue staining showed that AAV-KLF7 treatment (28.83 ± 2.44%) increased the percent of myelin sparing compared to the AAV-GFP treatment (23.92 ± 3.85%) (*t*_10_ = 2.636, *P* < 0.05) (Figures 7(b) and 7(e)).

### 3.7. AAV-KLF7 Significantly Infects DPSNs at T7 and T9 Levels after SCI as Detected by FG Retrograde Labeling

**Figure 8:**
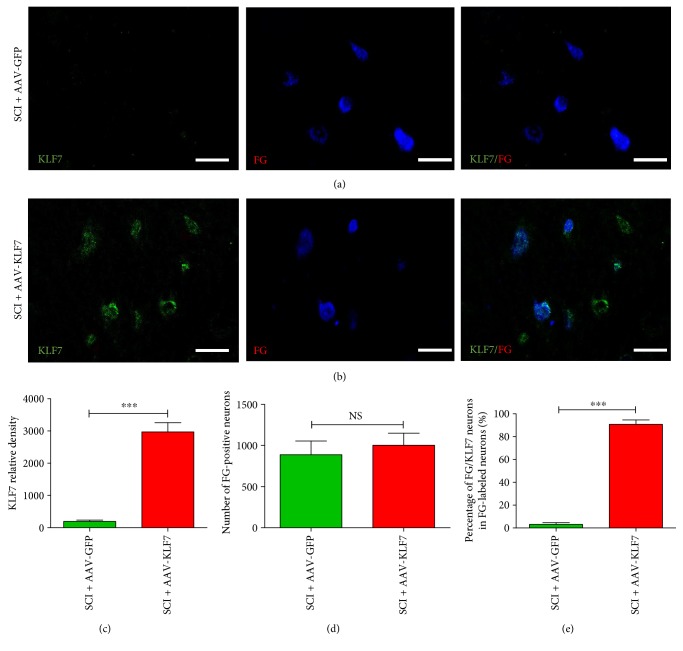
AAV-KLF7 significantly infects DPSNs at T7 and T9 levels after SCI. Five weeks after contusion injury, (a-b) representative immunohistochemical stainings for KLF7 (green) and FG (retrograde-labeled DPSNs, blue) in the Rexed lamina VII at the T7 and T9 levels are shown across the SCI + AAV-GFP and SCI + AAV-KLF7 groups. Cell counts were performed at the T7 and T9 levels with 15 sections per segment. Quantitative analyses of the KLF7 relative density (c), FG-labeled neurons (d), and percentage of FG/KLF7 double-labeled neurons (e) above the lesion area. *n* = 6 mice/group. Student's *t*-tests; green indicates SCI + AAV-GFP control; red indicates SCI + AAV-KLF7. ^∗∗∗^*P* < 0.001. NS: not significant. Scale bars: 50 *μ*m.

FG retrograde labeling of DPSNs was examined in Rexed lamina VII at T7–9 levels above the spinal cord lesion by immunofluorescence (Figures 8(a) and 8(b)). The results showed no difference in the number of FG retrograde-labeled neurons between the two groups (SCI + AAV-KLF7, 1003 ± 145.9; SCI + AAV-GFP, 887.7 ± 166.3; *t*_10_ = 1.277, *P* > 0.05) (Figure 8(d)). However, we found that KLF7 expression and thus the percentage of FG/KLF7 double-labeled neurons in the population were higher in the SCI + AAV-KLF7 group compared to the SCI + AAV-GFP group (KLF7: SCI + AAV-KLF7, 2971 ± 701.1; SCI + AAV-GFP, 195.1 ± 101.5; *t*_10_ = 9.598, *P* < 0.001; percentage of FG/KLF7 neurons: SCI + AAV-KLF7, 90.75 ± 3.84%; SCI + AAV-GFP, 3.11 ± 1.54%; *t*_10_ = 51.83, *P* < 0.001) (Figures 8(c) and 8(e)). These results suggest that while treatment did not impact the number of labeled DPSNs, the AAV-KLF7 treatment can effectively be transduced to DPSNs at the T7–T9 levels above the spinal cord lesion.

### 3.8. AAV-KLF7 Significantly Improves DPSN Plasticity around the T10 Lesion Site 5 Weeks following SCI

**Figure 9:**
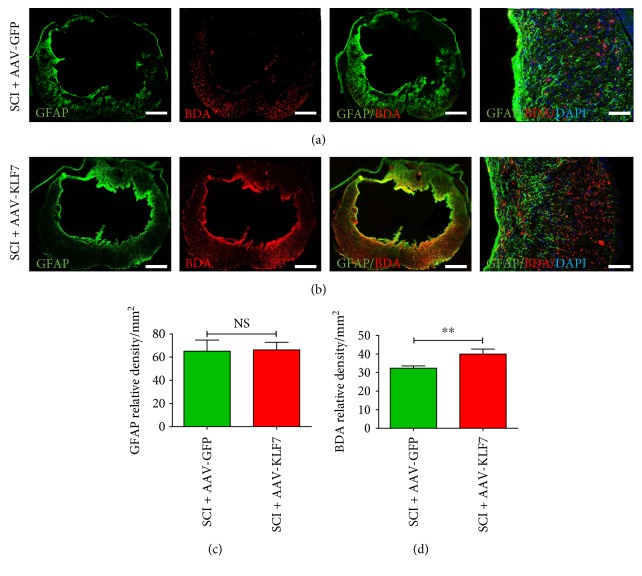
AAV-KLF7 significantly improves DPSN axonal plasticity around the lesion. Five weeks following contusion injury, (a-b) representative immunohistochemical stainings for GFAP (green) and BDA (labeling DPSN axons, red) and nuclear staining (DAPI, blue) surrounding the epicenter of SCI + AAV-GFP and SCI + AAV-KLF7 groups are presented. Quantitative analyses of the GFAP relative density (c) and BDA relative density (d) around the lesion area. *n* = 6 mice/group. Student's *t*-tests; green indicates SCI + AAV-GFP control; red indicates SCI + AAV-KLF7. ^∗∗^*P* < 0.01. NS: not significant. Scale bars: 300 *μ*m or 100 *μ*m.

The glial fibrillary acidic protein- (GFAP-) immunoreactive astrocytes and BDA-labeled DPSN axons and terminals were examined around the spinal cord lesion site by immunofluorescence staining (Figures 9(a) and 9(b)). The results showed no difference in GFAP expression between the two groups (SCI + AAV-KLF7, 66.31 ± 6.52; SCI + AAV-GFP, 65.14 ± 9.57; *t*_10_ = 0.234, *P* > 0.05) (Figure 9(c)); however, the number of BDA-labeled DPSN axons and terminals extended around the lesion through the spared white matter was significantly increased in the SCI + AAV-KLF7 group (39.95 ± 2.70) compared to the SCI + AAV-GFP group (32.37 ± 1.25) (*t*_10_ = 4.964, *P* < 0.01) (Figure 9(d)). These findings suggest that AAV-KLF7 treatment may promote DPSN plasticity at the lesion site.

### 3.9. AAV-KLF7 Promotes DPSN Axonal Plasticity and Synaptogenesis with Motor Neurons at the Caudal Spinal Cord following SCI

**Figure 10:**
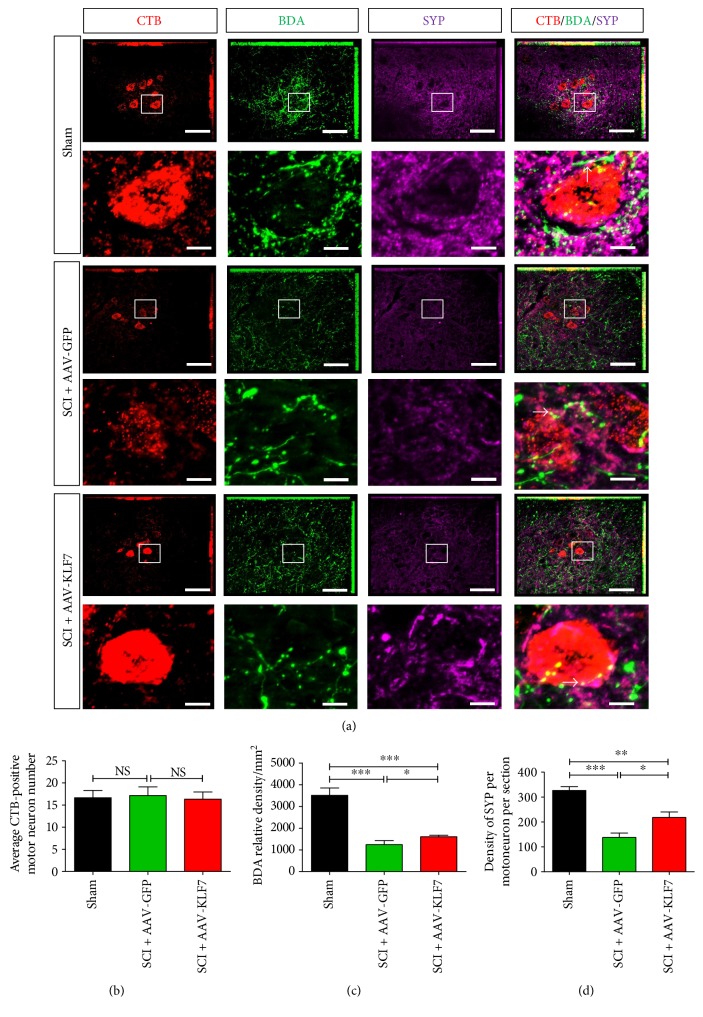
AAV-KLF7 promotes DPSN axonal plasticity and induced synapse formation with motor neurons at the caudal spinal cord. (a) Contact between motoneurons (CTB retrogradely labeled, red) and DPSN axons (BDA-labeled, green) and synapse formation (synaptophysin-labeled, purple) are shown. Block indicates the new synapses formation (white) between DPSN axons and motoneurons. Quantitative analyses of the motor neuron targeted CTB-labeled axons (b), BDA relative density (c), and SYP density per motoneuron per section (d) at the caudal spinal cord. *n* = 6 mice/group. ^∗^*P* < 0.05, ^∗∗^*P* < 0.01, and ^∗∗∗^*P* < 0.001. NS: not significant. One-way ANOVA, Tukey's post hoc test. Scale bars: 100 *μ*m.

Next, we performed immunohistochemical staining of DPST axons and terminals using BDA labeling in conjunction with retrograde CTB labeling of lumbar motor neurons and synaptic (SYP) protein detection at the caudal spinal cord (Figure 10(a)). We found that the expression of BDA and SYP was significantly decreased following SCI compared to that of uninjured sham controls; moreover, the expression of BDA and SYP per motoneuron in the SCI + AAV-KLF7 group was significantly higher than that in in the SCI + AAV-GFP group (BDA: SCI + AAV-KLF7, 1600 ± 74.28; SCI + AAV-GFP, 1238 ± 188.6; and sham, 3509 ± 342.0; *F*_2, 15_ = 197.8, *P* < 0.001; SYP per motoneuron: SCI + AAV-KLF7, 218.3 ± 61.66; SCI + AAV-GFP, 137.9 ± 49.79; and sham, 326.6 ± 43.13; *F*_2, 15_ = 26.45, *P* < 0.001) (Figures 10(c) and 10(d)). However, the number of CTB-labeled motoneurons at the caudal spinal cord showed no difference across the three groups (SCI + AAV-KLF7, 16.33 ± 1.633; SCI + AAV-GFP, 17.17 ± 1.941; and sham, 16.67 ± 1.633; *F*_2, 15_ = 0.348, *P* > 0.05) (Figure 10(b)). These results demonstrated that KLF7 upregulation enhances DPST plasticity at the caudal spinal cord following SCI.

### 3.10. AAV-KLF7 Promotes Axonal Myelination around the Lesion Site following SCI

**Figure 11:**
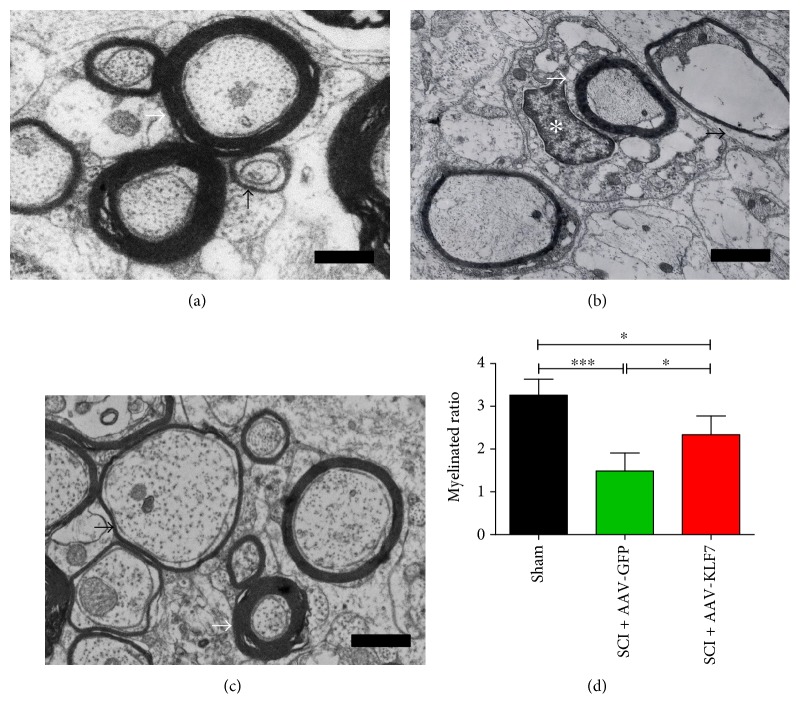
AAV-KLF7 promotes axon myelination around the lesion. Transmission electron microscope (TEM) analysis was used to assess remyelination of spinal axons across the sham (a), SCI + AAV-GFP (b), and SCI + AAV-KLF7 groups (c) (*n* = 10). Quantitative analyses of the myelinated ratio (d). ^∗^*P* < 0.05, ^∗∗∗^*P* < 0.001, one-way ANOVA, Tukey's post hoc test. Bar indicates 1 *μ*m.

Electron microscopy showed that the myelinated ratio of the uninjured sham group was higher than that of the injured SCI + AAV-KLF7 and SCI + AAV-GFP groups. Notably, the myelinated ratio in the SCI + AAV-KLF7 group was significantly higher than that in the SCI + AAV-GFP group (SCI + AAV-KLF7, 2.33 ± 0.438; SCI + AAV-GFP, 1.48 ± 0.42; and sham, 3.26 ± 0.37; *F*_2, 27_ = 18.62, *P* < 0.001) (Figures 11(a), 11(b), 11(c), and 11(d)). Though not totally in agreement, the results overall suggest that SCI + AAV-KLF7 improves axonal myelination around the lesion site.

### 3.11. KLF7 Expression in Oligodendrocyte Precursors after AAV-KLF7 Treatment following SCI

To determine the cellular localization of KLF7 expression after AAV-KLF7 treatment, the double-label immunohistochemistry for KLF7 and CC1 (labeling oligodendrocytes), NG2 (labeling oligodendrocyte precursors), and GFAP (labeling astrocytes) was performed.

**Figure 12:**
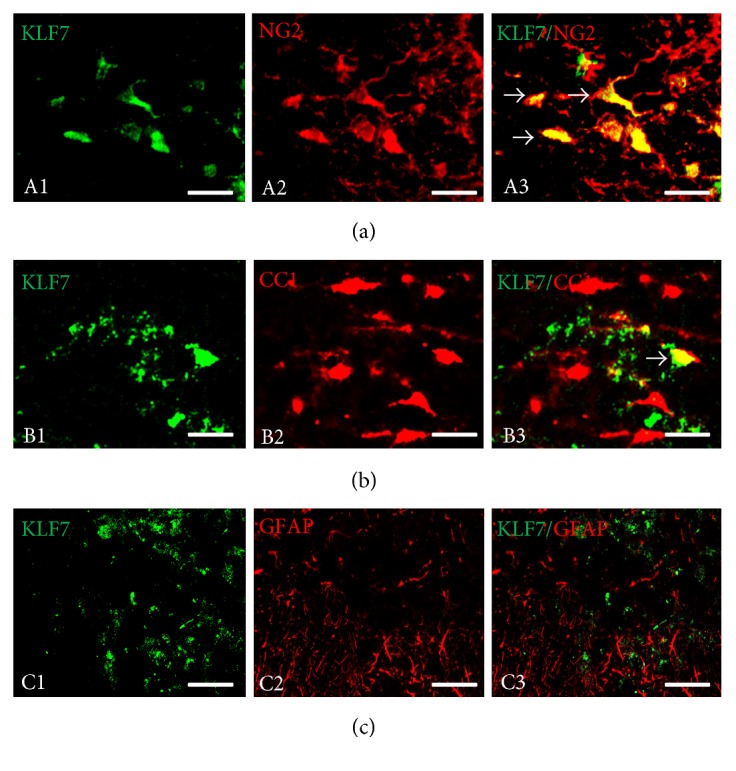
Cellular localization of KLF7 expression in the mouse spinal cord after spinal cord injury (SCI). (a) Little KLF7 immunoreactivity (IR) was localized in oligodendrocytes indicated by CC1 IR (arrows). (b) KLF7 immunoreactivity (IR) was localized in oligodendrocyte precursors indicated by NG2 IR (arrows). (c) No KLF7 immunoreactivity (IR) was localized in astrocytes indicated by GFAP IR (arrows). Scale bars: 100 *μ*m.

The majority of NG2-positive cells expressed KLF7; the percentage of NG2/KLF7 neurons is 84.22 ± 6.95% (Figure 12(a)). However, a few CC1-positive cells expressed KLF7 (percentage of CC1/KLF7 neurons: 10.23 ± 2.39%) (Figure 12(b)), and no astrocytes were found to express KLF7 (Figure 12(c)). The results overall suggest that overexpressed KLF7 in oligodendrocyte precursors cells may promote axonal remyelination after SCI.

### 3.12. AAV-KLF7 Infection Does Not Affect Tibialis Anterior Muscle Atrophy of Motor Endplate Density

**Figure 13:**
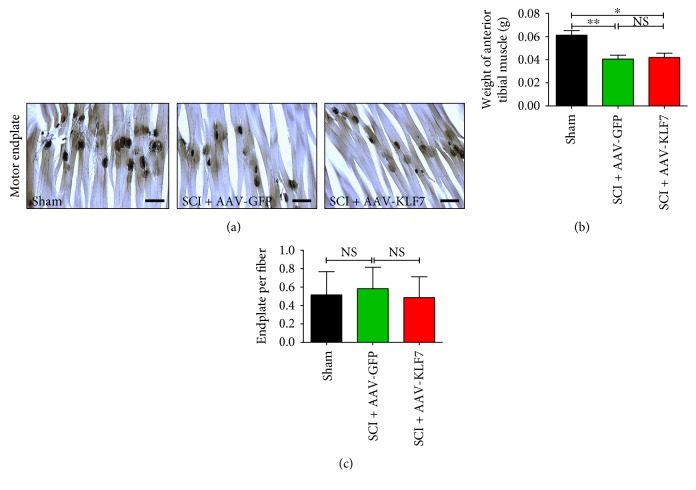
Weight of TA muscle and the number of motor endplate are unaffected by KLF7 following SCI. (a) Acetylcholinesterase staining in sham, SCI + AAV-GFP, and SCI + AAV-KLF7 groups. Quantitative analyses of TA muscle weight (b) and the numbers of endplate per fiber (c) (*n* = 6 mice/group). Error bars show SD. ^∗^*P* < 0.05, ^∗∗^*P* < 0.01, one-way ANOVA, Tukey's post hoc test. NS: not significant. Scale bars: 50 *μ*m.

To assess whether AAV-KLF7 infection had any effect on TA muscle atrophy, we assessed the muscle weight of TA and motor endplate density across the three groups. Following SCI, TA muscle weight is decreased by 34.42% as compared to that of the uninjured sham group; however, no difference was found in TA muscle weight between the SCI + AAV-KLF7 and SCI + AAV-GFP groups (SCI + AAV-KLF7, 0.041 ± 0.007 g; SCI + AAV-GFP, 0.040 ± 0.006 g; *t*_12_ = 0.299, *P* > 0.05) (Figure 13(b)). In addition, motor endplate staining showed no difference in the number of endplates per muscle fiber across either of the three groups (SCI + AAV-KLF7, 0.486 ± 0.225; SCI + AAV-GFP, 0.582 ± 0.232; and sham, 0.515 ± 0.251; *F*_2, 15_ = 0.262, *P* > 0.05) (Figures 13(a) and 13(c)).

### 3.13. KLF7 Transfection Significantly Enhances Motor Functional Recovery following SCI

**Figure 14:**
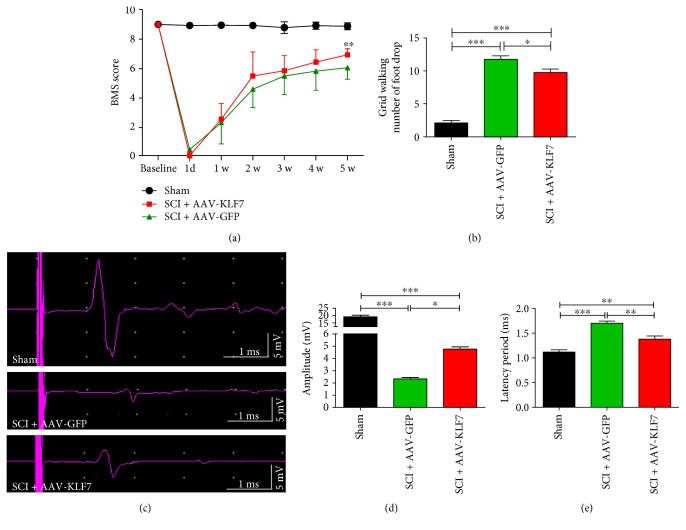
AAV-KLF7 significantly enhanced motor functional recovery following SCI. (a) Quantitative analyses of the Basso Mouse Scale (BMS) locomotor scores post-SCI (b), number of foot drops during grid walking five weeks postinjury (c), and tcMMEP responses five weeks postinjury (d), including the amplitude (mV) (e) and latency period (ms) from each group (*n* = 8), are shown. Error bars denote SD. ^∗^*P* < 0.05, ^∗∗^*P* < 0.01, and ^∗∗∗^*P* < 0.001, one-way ANOVA, Tukey's post hoc test.

BMS behavioral scores are represented in Figure 13(a); the results showed that BMS scores were significantly increased in the SCI + AAV-KLF7 group (6.93 ± 0.41) compared with the SCI + AAV-GFP group (6.06 ± 0.77) 5 weeks after injury (*F*_2, 21_ = 59.88, *P* < 0.001) (Figure 14(a)). In a similar vein, grid walk locomotor analysis showed that the number of foot drops during grid walking was significantly improved by AAV-KLF7 treatment (9.75 ± 1.48) compared to the AAV-GFP treatment (11.75 ± 1.48) (*F*_2, 21_ = 104.2, *P* < 0.001) (Figure 14(b)).

Finally, we measured electrophysiological characteristics of descending axons in the ventral spinal cord to assess functional recovery across the three groups (Figure 14(c)). The SCI + AAV-KLF7 (4.76 ± 0.44) and SCI + AAV-GFP groups (2.33 ± 0.28) displayed decreased wavelength amplitude compared to the uninjured sham group (19.17 ± 2.63) (*F*_2, 21_ = 205.7, *P* < 0.001) (Figure 14(d)); however, the SCI + AAV-KLF7 group displayed a significant improvement over the SCI + AAV-GFP group. Wavelength latency was increased in the injury groups compared to sham (SCI + AAV-KLF7, 1.38 ± 0.15; SCI + AAV-GFP, 1.70 ± 0.10; and sham, 1.11 ± 0.11; *F*_2, 21_ = 31.73, *P* < 0.001) (Figure 14(e)) though SCI + AAV-KLF7 significantly reduced this injury-induced latency. Overall, the SCI + AAV-KLF7 treatment showed a better electrophysiological response compared to the SCI + AAV-GFP treatment and supports a functionally relevant therapeutic impact of KLF7 overexpression.

## 4. Discussion

SCI induces a biphasic sequence whereby inhibitory factors targeting injured neurons and hindering recovery are followed by intrinsic repair elements such as sprouting and axonal plasticity. As investigators continue to unravel the complexities of the injury response, both inhibitory factors and endogenous repair targets have been exploited in preclinical models to optimize recovery. For example, high-throughput screening of inhibitory targets has revealed PTEN and Sac2 as inhibitors of axon plasticity in mouse cortical neurons [35], the first important step toward minimizing their influence postinjury. Conversely, a variety of targets implicated in endogenous repair mechanisms have been the source of manipulation studies, highlighting a complimentary approach to the optimization of post-SCI repair.

Due to the multifaceted nature of axonal plasticity, remyelination, and synapse formation (critical components of injury repair), targeting single genes for optimization is likely to yield limited improvements. Instead, many investigators have looked toward regulators of gene networks such as transcription factors of various regeneration-associated pathways. Here, we expanded upon previous reports of improved axonal regeneration and functional recovery attributed to the regulatory factor KLF7 established in the injured CST [10, 12, 36]. The goal of this study was to determine whether KLF7 treatment could be a novel strategy for DPSN axonal plasticity and functional repair after SCI.

Here, we confirm the effectiveness of KLF7 upregulation as a therapeutic strategy in descending propriospinal axon plasticity following contusive SCI. The plasticity and regenerative capacity of which are likely essential to transmission of supraspinal motor command and thereby an important factor for functional motor recovery [19].

Foremost, this study set out to characterize the effectiveness of AAV-driven upregulation of endogenous KLF7 and its downstream targets *in vitro*. We report that spinal cord neuron transfection was efficient and significantly upregulated the target elements. Particularly, KLF7 induction increased the expression of the neurotrophic factor NGF and its receptor TrkA, elements previously implicated in axon development and regeneration after injury, mainly through MAPK-ERK1/2 and PI3K pathways [37]. As predicted, the advent of upregulated KLF7 and NGF/TrkA was supported by increased neurite outgrowth observed in both spinal neurons and DRG explants. These results are in agreement with previous reports of KLF7 upregulation and axon growth in culture [38] and compliment earlier findings that KLF7 disruption is associated with deficits in neurite outgrowth and axonal misprojection [39].

After confirmation of AAV-KLF7 efficiency *in vitro*, we next applied the upregulation strategy in a live model of contusive T10 SCI. *In vivo*, the AAV-KLF7 construct was sufficient to drive KLF7 overexpression up to 5 weeks postinjury, prolonging the endogenous elevation of KLF7 by 1-2 weeks compared to the intrinsic injury profile. As observed in culture, several KLF7 targets and injury-related proteins were maintained in an elevated state 5 weeks after injury. Particularly NGF, the NGF receptor TrkA, and GAP43 all demonstrated some intrinsic, injury-related elevation which was enhanced significantly by AAV-KLF7. As noted earlier, the NGF/TrkA signaling cascade plays a role in axon regeneration after injury. Similarly, the growth-associated protein 43 (GAP43) has demonstrated importance in axonal regeneration [40], containing regeneration-specific proneural enhancer regions in fish homologs [41]. Complimentary to these observations, the injury-related response of the myelin protein P0 was significantly mitigated by AAV-KLF7 treatment. While injury induces a drastic drop in P0 abundance, likely representing demyelination and degeneration, AAV-KLF7 treatment demonstrated significant elevation of the protein compared to AAV-GFP treatment. Considering myelination assessments conducted via electron microscopy (further detailed below), there was corroborating evidence that KLF7 overexpression plays a role in myelin preservation and/or axon remyelination. Interestingly, closer assessment of KLF7-upregulating cells revealed that the majority of KLF7 upregulation was displayed by oligodendrocyte precursors (NG2^+^ cells). This appears to complement earlier reports that KLF6 plays a critical role in oligodendrocyte differentiation [42]and further substantiates the role of KFL7 in myelin preservation and remyelination after SCI.

Importantly, *in vivo*, pathological assessment revealed that KLF7 overexpression was not sufficient to influence the lesion area or volume; however, the treatment did significantly improve the amount of myelin sparing following contusive SCI. These findings echo a previous study in which combined KLF7 overexpression and chondroitinase were not sufficient to circumvent the lesion size [12]. Despite this restriction, myelin sparring is significant and has been correlated with improved oligodendrocyte survival and improvements in motor function in human spinal cord injury [43].

Wang et al. recently discuss that KLF7 upregulation may bear differential effects based on the cell type and injury model [12]. In this vein, it was important for our group to examine a population of neurons that has been previously highlighted in the preservation and recovery of motor function during SCI. The anatomical positioning and projection patterns of propriospinal neurons have made them the subject of an intense study in SCI. Proposed to be involved in the regulation of locomotion, limb coordination, and postural support, propriospinal neurons may even possess the capacity to circumvent denervation by relaying supraspinal motor commands to the lumbar spinal cord after thoracic injury [44]. Additionally, descending propriospinal neurons (DPSNs) in the ventral motor area could directly innervate motor neurons of hind limb muscles and in turn may be innervated by injury-mediated supraspinal sprouting, collectively positioning the neuron pool as a major role player in functional recovery following SCI [45]. Indeed, some apparently inherent features of propriospinal neurons compared to supraspinal counterparts reveal an enhanced resilience and injury-repair response in the form of sparring and sprouting [46, 47]. An important neuroprotective property of surviving propriospinal neurons which project past the lesion site is their ability to form a “bridge” between regenerated descending signals and their targets past the lesion [48]. These properties (coincidental with enhanced responsiveness to SCI therapeutic strategies [17]) suggest perhaps a more viable therapeutic approach toward recovery of motor function in SCI.

Examination of DPSN projections via retrograde labeling revealed that L2-projecting DPSNs were sensitive to KLF7 treatment, displaying increased expression of the transcription factor. This finding likely indicates that AAV-KLF7 is effective at targeting DPSNs *in vivo* and likely exerting its putative functions in that neuron pool, including neurotrophic factors and axonal growth cues as discussed above. Similarly, anterograde tracing via BDA labeling demonstrated that DPSN axons are increasingly extended around the lesion through the spared white matter in KLF7-treated groups. Taken together, increased presence of BDA-labeled DPSN tracts and increased SYN expression of motor neuron targets in the caudal spinal cord indicate plasticity and sprouting of spared axons and importantly suggest meaningful contributions to functional recovery. One discrepancy we found was the absence of injury or treatment effect in the number of retrogradely labeled neurons compared to the anterogradely labeled neurons. We suggest that this may be due to the fact that cell bodies of short propriospinal neurons above the lesion are relatively resistant to retrograde death and apoptosis after axotomy [49]. At present, there is no evidence that KLF7 overexpression promotes spinal cord neuron survival or inhibits cell apoptosis *in vitro*. However, *in vitro*, KLF7 overexpression promotes neurite outgrowth of spinal cord neurons and DRG explants. Therefore, *in vivo*, the action of KLF7 may occur at the cell soma or axon of these DPSNs to promote their axonal spouting across the SCI lesion but is perhaps not a major contributor to labeled DPSN cell survival or apoptosis. As such, further investigation is needed to reveal the effect and mechanism of KLF7 overexpression on neuron survival.

Examination of motor neurons and target muscle tissues identified that KLF7 treatment did not impact the number of motor neuron innervations of the sciatic nerve (identified by CTB labeling in the lumbar spinal cord), nor did it affect motor endplate densities or anterior tibial muscle weight. The apparent insensitivity of the lumbar motor neurons and target muscles likely reflects their distance from the T10 contusion injury site. Despite this, KLF7 treatment did impact functional outcomes as reported by behavioral and locomotor examination 5 weeks after injury. The reconciliation of improved motor capacity and restricted axonal plasticity observed at motor targets may support the previously described notion that functional recovery in SCI may be mediated by DPSN axonal bridging at the lesion site, communicating supraspinal motor commands to motor neurons. This argument is strengthened by the observed increase in synaptogenesis markers, the enhanced myelination of existing axons, and the improved conductivity of the DPSN axons of the ventral spinal cord. Effectively, AAV-KLF7 treatment after SCI appears to strengthen the regenerative and functional capacity of DPSNs through spared tissue, likely contributing to improved behavioral outcomes. This is significant particularly because previous reports have rejected the notion that KLF7 manipulation could independently contribute to functional recovery after SCI [12].

These results provide evidence that actively engaging KLF7 improves function and that one likely mechanism may be the upregulation of KLF7 in oligodendrocyte precursors and subsequent differentiation which may promote myelin sparring and/or remyelination which plays a critical role in functional recovery after SCI. Myelination of axons is a prerequisite for the rapid propagation of nerve impulses. To obtain adequate functional recovery, regenerated axons must be myelinated. Previous studies have confirmed that surviving oligodendrocyte precursors are the major source for oligodendrocyte (OL) replacement and remyelination after SCI [50]. Additionally, a recent study has confirmed the critical nature of the KLF7-relative KLF6 in the differentiation of oligodendrocyte precursors in the adult CNS [42]. Here, we propose that overexpression of KLF7 in oligodendrocyte precursors promotes OL replacement and meaningfully contributes to enhanced DPSN remyelination after SCI, promoting DPSN axonal plasticity and myelination and consequently promoting functional recovery. However, the conclusive evidence of this mechanism requires the design of a sequence of new experiments which is beyond the scope of the current study.

Another likely mechanism underlying the functional contributions of KLF7 upregulation is the reorganization and reengagement of rostrocaudal spinal interneuronal networks. Functionally, the propriospinal intersegmental linkage can mediate and/or facilitate motor coordination, as observed by several studies which have demonstrated that spared spinal pathways undergo extensive reorganization to increase recruitment of propriospinal pathways for motor recovery. Additionally, the short propriospinal neurons primarily located in T7–T9 which innervate lumbar motor neuronal pools may relay signals from the long supraspinal descending systems to the motor neurons in the lumbar cord, creating new functional circuits around an incomplete lesion and providing the substrate for locomotor recovery [49]. We believe that the rostrocaudal interneuronal networks are reengaged after KLF7 treatment to enhance locomotor recovery. This proposition is supported by greater axonal plasticity of propriospinal interneurons bilaterally at and below the level of the lesion site denoting enhanced contact of lumbar neuronal networks.

An important study by Siebert et al. recently examined the time-dependent gene expression changes in thoracic PNs following SCI. Three days after SCI (a complete transection at T9), thoracic PNs showed an upregulated expression of a suite of regeneration-associated genes (including Actb, Atf3, Cd44, Crem, Ctsb, Gap43, Hsbp1, Jun, Pacap, Sox11, Stat3, and Tubb3). Some of these genes play important roles in the production of injury-associated mRNA transcripts, while others can affect processes such as growth cone dynamics [51, 52]. In our study, AAV-KLF7 treatments exerted their plastic/regenerative effects by acting on the DPSN body. Thoracic DPSN showed an upregulated expression of KLF7, an important regulator of growth factors such as NGF and TrkA which promote sprouting and axon regeneration, a key feature of path finding and new synapse formation. Evidence of this interaction can be deduced from the enhanced neurite outgrowth which was observed in response to KLF7 *in vitro* and via axon plasticity in DPSN populations with a sustained effect. DPSN sprouting in turn may facilitate functional recovery via one of its many structural and physiological intersections with motor signaling and command.

Despite the wealth of knowledge on manipulations that enhance DPSN axonal sprouting and consequent motor functional recovery, the intrinsic mechanisms underlying recovery have not yet been fully elucidated. Further investigation is needed to reveal the broader mechanisms of these changes in plasticity and how they recognize specific partners to form synapses. Understanding the underlying molecular mechanisms may be helpful in establishing new therapeutic methods for the recovery of impaired function. Here, the proposed involvement of KLF7 and downstream growth factors may provide more insight than has previously been put forth. Our observed KLF7 upregulation falls in line with previous findings which reported the time-dependent gene expression changes in thoracic PNs following SCI. For example, after SCI, thoracic PNs showed an upregulated expression of a suite of regeneration-associated genes; some of which play important roles in the production of injury-associated mRNA transcripts, while others can affect processes such as growth cone dynamics [51, 52]. For example, Gap43 expression in neurons has been correlated with axonal outgrowth, path finding, and new synapse formation via interactions with extrinsic signaling molecules (such as neurotrophic factors and cell adhesion proteins) and intrinsic cytoskeletal elements including actin [53, 54]. KLF7, in a similar vein, may underlie neurophysiological and functional recovery.

Another important consideration is the assumption that increased KLF7-mediated growth signaling is exclusively a beneficial parameter. The reality is that growth factor signaling is a multifaceted business and, though typically seen as therapeutic in neurodegeneration, cannot be conclusively accepted as unilaterally beneficial without rigorous testing. Grounds for the presumed therapeutic nature of growth factors come from the existence of neurotrophic factors as concentration gradients directing axonal growth toward their targets. However, growth factor concentration may also influence misguidance of axonal targets and negatively impact functional neurocircuitry [55]. Similarly, some growth factors, including the KLF7-mediated Trk receptors, have shown antagonizing action on cell survival in certain contexts [56, 57]. Considering the growing complexity of ligands, associated factors, and signaling cascades, it would not be surprising if additional layers of interactions impinge upon the overall contributions of the growth cues. As such, future studies will have to tease out the contributions of enhanced growth and neurotrophic factors in the context of cell type, spatial and temporal patterning, and interaction with the insult [58, 59].

## 5. Conclusion

The intrinsic regulatory capacity of KLF7 in axonal growth and synaptogenesis can be manipulated to overcome injury-related restrictions in a model of T10 contusive SCI. Likely operating under a variety of neurotrophic, survival, and myelinogenesis pathways, we have identified a novel population of KLF7-responsive spinal cord neurons. Here, we characterized the extent of KLF7-driven enhancements and limitations on the DPSN axons, outlining axonal plasticity, synapse formation, and functional parameters which are likely accountable for observed functional recovery. We conclude that KLF7 overexpression is stable and functional and targets a population of spinal neurons heavily implicated in motor regulation. These DPSNs respond in kind by strengthening their functional capacity postinjury, likely contributing to overall improved behavior. In closing, this study supports the optimization of the intrinsic injury response as an effective therapeutic strategy for SCI treatment. And while other targets and strategies continue to be elucidated, investigators will be able to develop improved combinatorial strategies to target the multiple deficits and enhance the multiple repair signals of the SCI response, bringing the field ever closer to optimal SCI recovery.

## Abbreviations

AAV: Adeno-associated virus [2]
TA: Anterior tibial
BDA: Biotinylated dextran amine
BMS: Basso Mouse Scale
CNS: Central nervous system
CST: Corticospinal tract
CTB: Cholera toxin B
d: Day
DRG: Dorsal root ganglia
DPSNs: Descending propriospinal neurons
FG: Fluoro-Gold
GAP43: Growth-associated protein
GFAP: Glial fibrillary acidic protein
KLF7: Krüppel-like factor 7 [1]
NGF: Nerve growth factor [3]
OL: Oligodendrocytes
P0: Peripheral myelin protein zero
PNs: Propriospinal neurons
SCI: Spinal cord injury
tcMMEP: Transcranial magnetic motor-evoked potentials
TEM: Transmission electron microscope
TrkA: Tyrosine kinase A
w: Week.
